# Preliminary study on mercury pollution affecting soil bacteria near a mercury mining area

**DOI:** 10.3389/fmicb.2025.1539059

**Published:** 2025-02-07

**Authors:** Jianxiong Du, Yili Yuan, Jianfeng Li, Shuqing Zhang, Yuxiang Ren

**Affiliations:** ^1^School of Management Science and Engineering, Guizhou University of Finance and Economics, Guiyang, China; ^2^Key Laboratory of Biological Resources Exploitation and Utilization in Universities of Guizhou, Guizhou Education University, Guiyang, China; ^3^School of Foreign Languages, Guizhou Normal University, Guiyang, China

**Keywords:** bacterial community, mercury contamination of soil, phylum level, class level, stress, microbial dominance

## Abstract

In order to further explore the effect of mercury contamination soil (nearly 20 years) near a mercury mining area (Tongren, Guizhou, China) on the diversity and structure of the soil bacterial communities, five groups of soil samples (SMO2, SMO20, SMO30, SMO500, and SMO650) were collected at distances of 2, 20, 30, 500, and 650 m, respectively, from the only sewage outlet of a mercury mining area (Guizhou, China). All soil samples were collected from the 0–20 cm topsoil layer. After processing them, the soil microbial DNA was extracted from each soil sample, and sequenced via high-throughput sequencing technology. The sequencing results indicated a significantly greater diversity of the soil bacterial community in SMO2, SMO20, and SMO650 (relative high mercury contents) than in SMO300 and SMO500 (relative low mercury contents). Alpha diversity analysis revealed that the soil bacterial community diversity in SMO2 and SMO20 significantly exceeded that in SMO30, SMO500, and SMO650. The soil bacterial community structure analysis revealed identical and distinct dominant bacterial communities within the soil sample groups at both phylum and class levels. According to the further analyzed relationships between the soil environmental factors and bacterial community abundance for each sample group, the pH, distance (mercury content), and electrical conductivity (EC) had greater impacts on the structure of the soil bacterial community than available N, P, K. The survival of high relative abundance bacterial community taxa in the microbial communities provides compelling evidence of the high adaptability of bacteria to long-term mercury contamination of the soil environment. The results of this study provide a scientific reference and impetus for further research on the mechanism(s) responsible for tolerance to high mercury stress in mercury-contaminated soil.

## 1 Introduction

The health of soil ecosystem is critical, being indispensable for food security and providing a living environment for the propagation, development, and growth of most microorganisms (Asadul et al., 2024; Antonio et al., 2024). Stable and good soil quality is essential for all types of biological communities to maintain a relatively stable living state (Remya and Suja, 2024; Chen et al., 2024; Liu K. K. et al., 2024). Soil links the atmosphere and water, and so atmospheric pollutants and aquatic pollutants can easily enter soil, resulting in soil pollution. Compared with the atmosphere and water, soil contains about 90% of environmental pollutants worldwide. The common heavy metals causing soil pollution mainly include mercury, cadmium, chromium, lead, arsenic, zinc, copper, and nickel (Wang et al., 2024; Tang et al., 2024; Liu S. C. et al., 2024; Wen et al., 2024; Li et al., 2024a; Khan et al., 2024).

Mercury is a heavy metal that has been identified as a key controlled pollutant among 129 priority control pollutants (Khairun et al., 2022; Tao et al., 2022; Zhao J. T. et al., 2014). In recent years, soil mercury contamination has occurred worldwide, seriously affecting the soil quality and soil ecosystems (Xia et al., 2022; Senila et al., 2023; Paiva et al., 2024; Maha et al., 2024; Nuryanty et al., 2024). Once mercury enters the soil, under the action of δ-Proteobacteria such as sulfate-reducing bacteria and iron-reducing bacteria, Hg^2+^ can be converted into the more toxic methylmercury, and transferred through crops, and then bioaccumulation in animals and humans via various food chains, all of which causes a suite of ecological and environmental problems (Melissa et al., 2023; Uwiringiyimana et al., 2023). The effects of heavy metal pollution on soil microorganisms have been reported (Li et al., 2024b; Xu, 2008), including mercury’s impact on soil microorganisms (Lavezzo et al., 2020; Liu et al., 2018; Giovanella et al., 2016).

To further explore the effect of mercury contamination of soil near mercury mining areas on bacterial communities under natural environmental conditions, in this study we collected samples from the 0–20 cm topsoil layer at different distances from the only outlet near an abandoned mercury mining area in Tongren, Guizhou Province, China. Then, high-throughput sequencing was used to explore the effects of mercury contamination of soil on soil bacterial community diversity and structures. These results provide some insights into the effects of mercury on bacterial communities and also provide support for further exploration of mercury-resistant bacterial communities and plants to remediate mercury-contaminated soils.

## 2 Materials and methods

### 2.1 Basic overview of the study area

**FIGURE 1:**
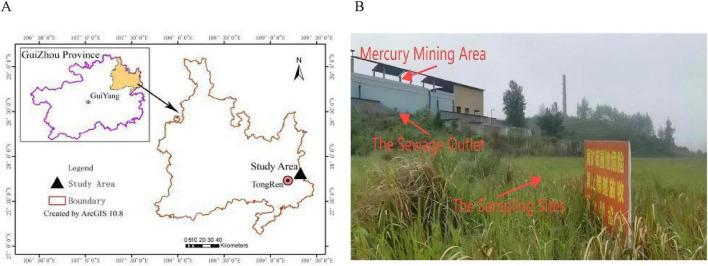
**(A)** The map of the study area. **(B)** The map of sampling sites.

The study area (109^°^07′–109^°^24′E; 27^°^24′–27^°^38′ N) is located in Tongren in the eastern part of Guizhou Province, China (Figures 1A, B). The landforms include low mountains, hills, and valleys. The soil type is brown earth. The annual average temperature is about 13–14^°^C. It is rich in mineral resources and contains the highest mercury reserves in China. However, due to a lack of resources, its chief mercury mine was closed by the local government in 2001. The map in Figure 1A was made in ArcGIS software (v10.8, United States).

### 2.2 Soil sampling

All soil samples were collected from the area surrounding the abandoned mercury mining area in Tongren, Guizhou Province, China. The soil samples were collected on 9 May 2022. Five groups of soil samples were collected from the only sewage outlet; the sewage outlet was taken as the center of the circle; the SMO2 (the four replicates numbered MSO1, MSO2, MSO3, and MSO4), SMO20 (CMSO21, CMSO22, CMSO23, and CMSO24), SMO30 (GMSO301, GMSO302, GMSO303, and GMSO304), SMO500 (GMSO500, GMSO501, GMSO502, and GMSO503), and SMO650 (GMSO651, GMSO652, GMSO653, and GMSO654) groups of soil samples were collected at 2, 20, 30, 500, and 650 m from the sewage outlet, respectively. The four replicates of each group of soil samples were collected at different interval positions with the same radius. All soil samples were collected from the 0–20 cm topsoil layer.

The soil samples were crushed, and impurities were removed. Next, the soil samples was sieved through a 1 mm mesh. Each soil sample was divided into two parts: one for measuring the soil properties (natural curing, temperature 25–35^°^C, relative humidity 20–60^°^C) and another for extracting the microbial deoxyribonucleic acid (DNA) (stored at −20^°^C).

### 2.3 Soil properties

The properties of the five groups of soil samples are presented in Table 1. The mercury content was determined via the cold atomic absorption method, by using a cold atomic absorption mercury analyzer (F732-V, China) (Qian, 2018); The detection limit of the instrument was 0.05 μg/L. pH, EC was determined via pH Meter (HANNA-HI98107, Italy) and Portable soil conductivity meter (EC-450, United States), respectively; The available N, P, K was determined via Alkaline hydrolysis diffusion method, Molybdenum antimony resistance colorimetric method, Flame photometric method, respectively (Yang et al., 2022).

**TABLE 1:** The properties of the five groups of soil samples (Modified from Du et al., 2023).

Groups of soil samples		Distance from sewage outlet (m)	AN (mg/kg)		AP (mg/kg)		AK (mg/kg)		EC (μ s/cm)	pH	Hg^2+^ (mg/kg)	
SMO2	MSO1	0.13	9.06	9.04 ± 0.06	11.14	11.06 ± 0.07	29.21	29.24 ± 1.21	122	7.5	147.04	140.45 ± 15.56
	MSO2	0.16	9.11		11.02		30.06		124	7.5	139.23	
	MSO3	0.18	8.98		11.08		30.14		125	7.5	119.51	
	MSO4	0.22	9.02		10.99		27.54		123	7.5	156.03	
SMO20	CMSO1	21.2	8.07	8.03 ± 0.07	10.34	10.43 ± 0.41	27.24	27.70 ± 0.49	112	8.0	167.14	152.44 ± 22.22
	CMSO2	20.10	8.01		10.13		27.31		114	8.0	139.25	
	CMSO3	19.60	7.94		10.22		28.07		110	8.0	128.33	
	CMSO4	20.32	8.11		11.04		28.16		113	8.5	175.02	
SMO30	GMSO301	30.50	8.97	8.63 ± 0.46	12.03	11.69 ± 0.45	30.04	29.29 ± 1.16	119	7.5	69.15	63.48 ± 11.18
	GMSO302	30.20	9.07		12.10		30.12		122	8.0	72.31	
	GMSO303	29.80	8.31		11.43		29.36		121	7.5	47.29	
	GMSO304	30.10	8.16		11.19		27.62		126	7.5	65.18	
SMO500	GMSO500	500.20	8.09	8.37 ± 0.44	10.21	10.67 ± 0.47	30.02	29.16 ± 1.03	114	8.5	61.51	59.77 ± 11.34
	GMSO501	500	8.21		10.32		28.22		123	8.5	45.42	
	GMSO502	500.10	8.15		11.03		28.31		116	8.5	73.06	
	GMSO503	499.80	9.02		11.11		30.07		126	8.5	59.10	
SMO650	GMSO651	650.30	9.11	8.88 ± 0.34	11.39	11.90 ± 0.34	29.11	28.57 ± 0.80	123	6.5	96.32	109.44 ± 12.35
	GMSO652	650.20	8.98		12.01		28.52		120	7.0	112.04	
	GMSO653	650.40	9.05		12.14		29.19		126	6.5	125.27	
	GMSO654	650.10	8.37		12.06		27.45		124	7.0	104.13	

Distance, AN, AP, AK, EC, pH, and Hg^2+^ denote the distance from the sewage outlet of the mercury mining area, available nitrogen, available phosphorus, available potassium, electrical conductivity, pH value, and Hg^2+^ concentration of the soil, respectively. The data are the mean ± standard deviation.

The available N (AN) content ranges from 8.03 to 9.04 mg/kg, the available P (AP) contents ranges from 10.43 to 11.90 mg/kg, the available K (AK) contents ranges from 27.70 to 29.29 mg/kg. The soil electrical conductivity (EC) ranges from 112 to 126, and the soil pH ranges from 6.5 to 8.5. The order of the Hg^2+^ contents are as follows: SMO20 > SMO2 > SMO650 > SMO30 > SMO500. The Hg^2+^ contents of SMO2, SMO20, and SMO650 are much higher than those of SMO30 and SMO500.

### 2.4 DNA extraction

The E.Z.N.A™ Mag-Bind Soil DNA Kit (Omega, M5635-02, United States) was used to perform the total community genomic DNA extraction, by following the manufacturer’s instructions. To measure the DNA concentration, Qubit 4.0 (Thermo, United States) was used to extract adequate amounts of high-quality genomic DNA.

### 2.5 16S rRNA gene amplification via PCR

Hypervariable regions V3–V4 of the bacterial 16S ribosomal ribonucleic acid (rRNA) gene were chosen as our target. After the DNA was extracted, polymerase chain reaction (PCR) amplification was immediately conducted. 2x Hieff^®^ Robust PCR Master Mix (Yeasen, 10105ES03, China) was used to amplify the 16S rRNA V3–V4 amplicon. Two universal bacterial 16S rRNA gene amplicon PCR primers (PAGE purified) were used: CCTACGGGNGGCWGCAG as the amplicon PCR forward primer and GACTACHVGGGTATCTAATCC as the amplicon PCR reverse primer.

### 2.6 16S gene library preparation, quantification, and sequencing

Hieff NGS™ DNA Selection Beads (Yeasen, 10105ES03, China) were used to purify the free primers and primer dimer species in the amplicon product. Samples were delivered to Sangon BioTech (Shanghai) for library preparation using Universal Illumina adapter and index. The Illumina MiSeq PE3 system (Illumina MiSeq, United States) was used to perform the sequencing following the manufacturer’s instructions.

### 2.7 Sequence processing, OTU clustering, representative tag alignment, and biological classification

After the sequencing was completed, two short Illumina readings were concatenated using PEAR software (v0.9.8) according to the overlap, and the fastq files were processed to generate separate fasta and qual files, which were then analyzed using standard methods. Usearch software (v11.0.667) was used to cluster the effective tags into operational taxonomic units (OTUs) with a similarity threshold of ≥ 97%. After removing the chimeric sequences and single-copy OTUs (with only one read), the remaining sequences were sorted into each sample based on the OTUs. The tag sequence with the highest abundance within each cluster was selected as the representative sequence. Taxonomic classification of the bacterial OTU representative sequences was performed by comparing them to the Ribosomal Database Project (RDP).

### 2.8 Statistical analysis

All alpha diversity indices were calculated using the Mothur software (version 3.8.31). The ANOVA test was used to calculated the multiple group sample (alpha) diversity. R vegan package (version 2.5-6) was used for redundancy analysis (RDA).

## 3 Results

### 3.1 Soil bacterial community diversity at the phylum level

**FIGURE 2:**
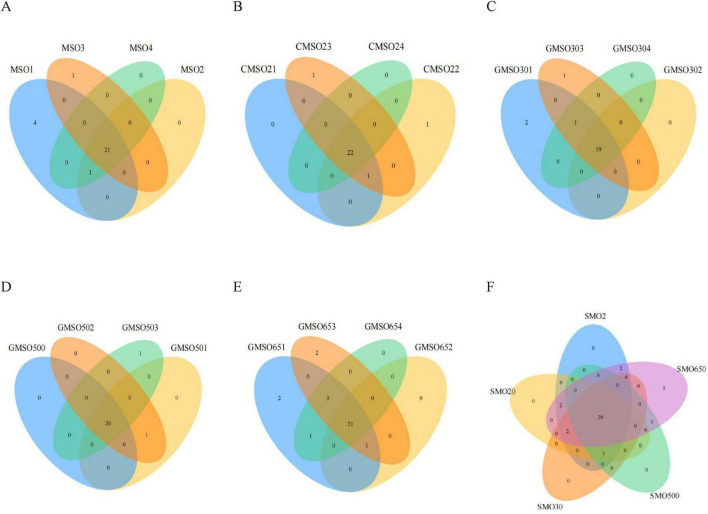
**(A)** The operational taxonomic units (OTUs) number of SMO2 group at the phylum level. **(B)** The OTUs number of SMO20 group at the phylum level. **(C)** The OTUs number of SMO30 group at the phylum level. **(D)** The OTUs number of SMO500 group at the phylum level. **(E)** The OTUs number of SMO650 group at the phylum level. **(F)** The OTUs number among the five different groups at the phylum level.

At the phylum level, through a clustering analysis of the OTUs of the sample sequences (at the 97% similarity level), we created Venn diagrams (Figures 2A–F). A total of 27, 25, 23, 22, and 31 OTUs were identified in the SMO2 group (Figure 2A), SMO20 group (Figure 2B), SMO30 group (Figure 2C), SMO500 group (Figure 2D), and SMO650 group (Figure 2E), respectively. Our results indicated that the OTUs number of the SMO2, SMO20, and SMO650 groups (high mercury content) was higher than that of the SMO30 and SMO500 groups (low mercury content). To sum up, the results of the within-group analysis indicated no significant differences in OTUs number among the four replicates of the same sample group at the phylum level.

The results of inter-group analysis of five groups (different mercury contents) are shown in Figure 2F. In total, 33 OTUs were identified in five groups. A total of 20 common OTUs were identified in five groups, only five unique OTUs in SMO650 group, and no unique OTUs in the other four groups. In summary, the results of inter-group analysis indicated significant differences in OTUs number of SMO650 group at the phylum level.

### 3.2 Soil bacterial community diversity at the class level

**FIGURE 3:**
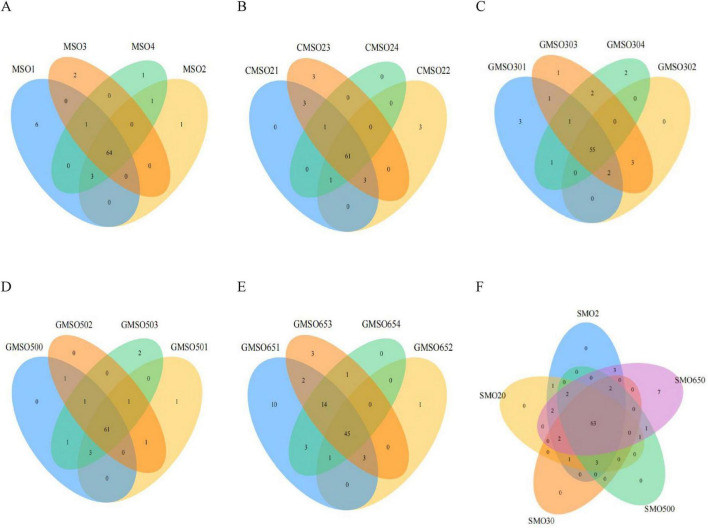
**(A)** The operational taxonomic units (OTUs) number of SMO2 group at the class level. **(B)** The OTUs number of SMO20 group at the class level. **(C)** The OTUs number of SMO30 group at the class level. **(D)** The OTUs number of SMO500 group at the class level. **(E)** The OTUs number of SMO650 group at the class level. **(F)** The OTUs number among five different groups at the class level.

At the class level, through a clustering analysis of the OTUs of the sample sequences at the 97% similarity level, we created a Venn diagram (Figures 3A–F). A total of 79, 75, 71, 72, and 83 OTUs were identified in SMO2 group (Figure 3A), SMO20 group (Figure 3B), SMO30 group (Figure 3C), SMO500 group (Figure 3D), and SMO650 group, respectively (Figure 3E). A highly similar trend in the results was found at the phylum level. Our results again indicated that the OTUs number of SMO2, SMO20, and SMO650 groups (high mercury content) was higher than that of SMO30 and SMO500 groups (low mercury content) at the class level. There were some differences in the OTUs number among its four replicates for SMO2, SMO20, SMO30, and SMO650 groups; no significant difference was found in the OTUs number among its four replicates for SMO500 group.

The results of inter-group analysis among five groups (different mercury contents) are shown in Figure 3F. In total, 88 OTUs were identified in five groups. A total of 63 common OTUs were identified in five groups, only seven unique OTUs in SMO650 group, and zero unique OTUs in the other four groups. In summary, the results of this inter-group analysis indicated significant differences in the OTUs number only SMO650 group at the class level.

### 3.3 Alpha diversity analysis

**FIGURE 4:**
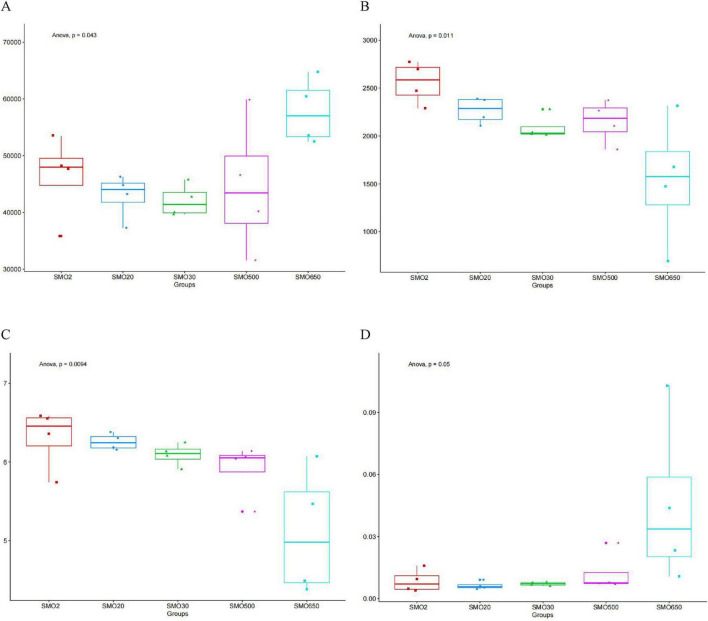
**(A)** The Reads of five different groups. **(B)** The operational taxonomic units (OTUs) of five different groups. **(C)** The Shannon index of five different groups. **(D)** The Simpson index of five different groups.

The alpha diversity indices of the bacterial communities in five groups were shown in the Figures 4A–D. The number of reads in five groups from high to low was SMO650 > SMO2 > SMO500 > SMO20 > SMO30 (Figure 4A). The OUTs number of five groups from high to low was SMO2 > SMO20 > SMO500 > SMO30 > SMO650 (Figure 4B). The Shannon index values of five different groups from high to low was SMO2 > SMO20 > SMO30 > SMO500 > SMO650 (Figure 4C). The Simpson index values of five different groups from high to low was SMO500 > SMO650 > SMO2 > SMO30 > SMO20 (Figure 4D). Anova test results among five different groups indicated significant difference in Reads, OUTs, Shannon index (*P* < 0.05). Comprehensive judgment based on the results of above four indices, the alpha diversity indices of the bacterial communities of five different groups was SMO2 > SMO20 > SMO30 > SMO500 > SMO650.

### 3.4 Soil bacterial community structure at the phylum level

**FIGURE 5:**
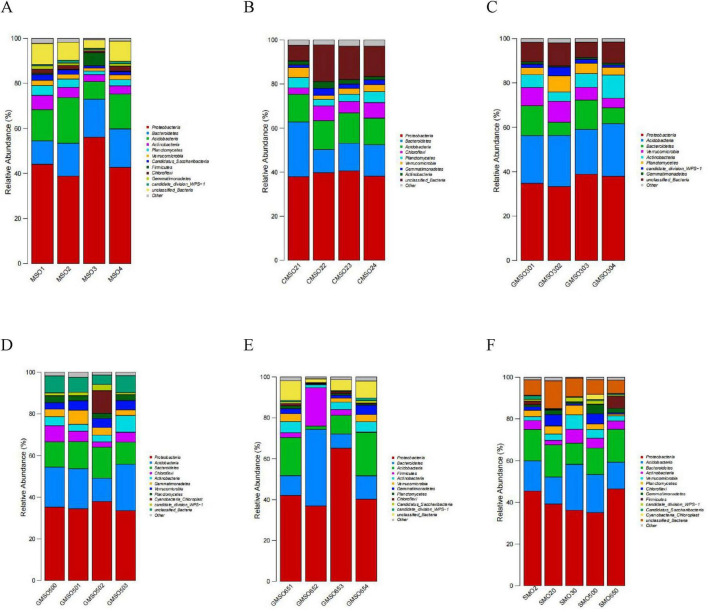
**(A)** The soil bacterial community relative abundance of SMO2 group at the phylum level. **(B)** The soil bacterial community relative abundance of SMO20 group at the phylum level. **(C)** The soil bacterial community relative abundance of SMO30 group at the phylum level. **(D)** The soil bacterial community relative abundance of SMO500 group at the phylum level. **(E)** The soil bacterial community relative abundance of SMO650 group at the phylum level. **(F)** The soil bacterial community relative abundances of five different groups at the phylum level.

At the phylum level, the relative abundances of the soil bacterial community members in SMO2 group (under 140.45 mg/kg Hg^2+^ stress) are shown in Figure 5A. Evidently, *Proteobacteria* (38.83–56.08%), *Bacteroidetes* (10.40–17.07%), *Acidobacteria* (7.83–20.26%), and *Actinobacteria* (3.12–6.30%) have very high relative abundances in four replicates. *Planctomycetes* (1.57–4.37%), *Verrucomicrobia* (1.34–2.29%), and *Candidatus_Saccharibacteria* (1.01–2.58%) also have high relative abundances in four replicates. The bacterial relative abundances in SMO20 group (under 152.44 mg/kg Hg^2+^ stress) are shown in Figure 5B. *Proteobacteria* (37.96–40.61%), *Bacteroidetes* (10.45–24.79%), *Acidobacteria* (12.06–13.78%), *Chloroflexi* (2.92–7.02%), and *Planctomycetes* (2.95–4.96%) have very high relative abundances in four replicates. *Verrucomicrobia* (1.85–4.57%), *Gemmatimonadetes* (1.17–3.18%), *Actinobacteria* (1.46–3.03%) have high relative abundances in four replicates. The bacterial relative abundances in SMO30 group (under 63.48 mg/kg Hg^2+^ stress) are shown in Figure 5C. *Proteobacteria* (33.28–38.85%), *Acidobacteria* (20.19–23.69%), *Bacteroidetes* (6.03–13.47%), *Verrucomicrobia* (4.30–9.38%), *Actinobacteria* (4.25–10.50%), *Planctomycetes* (3.11–7.26%), and *candidate_division_WPS-1* (1.10–4.00%) have very high relative abundances in four replicates. The bacterial relative abundances in SMO500 group (under 59.77 mg/kg Hg^2+^ stress) are shown in Figure 5D. *Proteobacteria* (33.59–37.96%), *Acidobacteria* (11.13–22.16%), *Bacteroidetes* (10.73–14.95%), *Chloroflexi* (2.52–7.69%), *Actinobacteria* (3.19–8.07%), *Gemmatimonadetes* (2.71–6.80%), *Verrucomicrobia* (3.10–4.53%), and *Planctomycetes* (2.40–3.38%) have very high relative abundances in four replicates. The bacterial relative abundances in SMO650 group (under 109.44 mg/kg Hg^2+^ stress) are shown in Figure 5E. *Proteobacteria* (36.93–65.18%), *Bacteroidetes* (6.94–37.46%), and *Acidobacteria* (1.57–21.12%) have very high relative abundances in four replicates. *Firmicutes* (0.28–18.80%), *Actinobacteria* (1.42–5.52%), *Verrucomicrobia* (0.23–3.86%), and *Gemmatimonadetes* (0.26–4.50%) have high relative abundances in four replicates. Overall, these within-group analysis results revealed some differences in the structure of the higher relative abundance of the soil bacterial communities of four replicates in the same group. In addition, the same soil bacterial community harbored different relative abundances of taxa in four replicates of a given sample group.

The soil bacterial community relative abundances in five groups (different Hg^2+^ contents) are shown in Figure 5F. *Proteobacteria* (35.17–46.49%), *Acidobacteria* (12.77–22.04%), and *Bacteroidetes* (10.16–15.80%) have extremely high relative abundances in five groups. *Proteobacteria* and *Bacteroidetes* have much higher relative abundances under a high Hg^2+^ press than under a low Hg^2+^ press; however, the relative abundance of *Acidobacteria* is the opposite. *Actinobacteria* (2.10–6.65%), *Verrucomicrobia* (1.93–6.94%), *Planctomycetes* (0.79–4.56%), *Chloroflexi* (0.68–5.41%), and *Gemmatimonadetes* (0.78–4.47%) also have high relative abundances in five groups, but *Actinobacteria* and *Verrucomicrobia* have much higher relative abundances under a low Hg^2+^ than under a high Hg^2+^. This inter-group analysis results indicated that the same and different taxa dominated the bacterial communities in five different groups (different Hg^2+^ contents). In addition, the same dominant bacterial taxa can have significant different relative abundances in five different groups (different Hg^2+^ contents) at the phylum level.

### 3.5 The soil bacterial community structure at the class level

**FIGURE 6:**
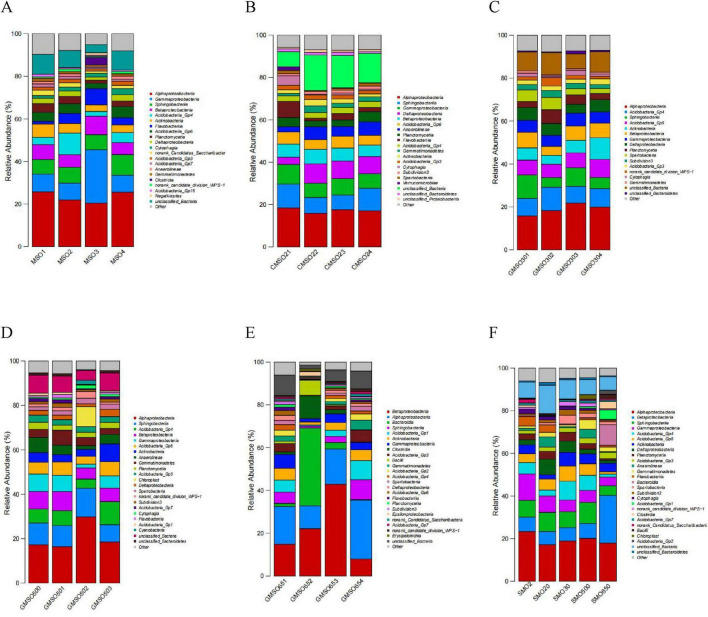
**(A)** The soil bacterial community relative abundance of SMO2 group at the class level. **(B)** The soil bacterial community relative abundance of SMO20 group at the class level. **(C)** The soil bacterial community relative abundance of SMO30 group at the class level. **(D)** The soil bacterial community relative abundance of SMO500 group at the class level. **(E)** The soil bacterial community relative abundance of SMO650 group at the class level. **(F)** The soil bacterial community relative abundances of five different groups at the class level.

At the class level, the relative abundances of soil bacterial community members in SMO2 group (with 140.45 mg/kg Hg^2+^) are shown in Figure 6A. *Alphaproteobacteria* (20.47–25.82%), *Gammaproteobacteria* (7.93–25.07%) and *Sphingobacteriia* (6.91–9.75%), *Betaproteobacteria* (5.56–8.71%), *Acidobacteria_Gp4* (3.09–6.20%), *Acidobacteria_Gp6* (2.50–5.20%), and *Actinobacteria* (2.21–10.16%) have very high relative abundances in four replicates. *Flavobacteriia* (1.28–7.62%), *Planctomycetia* (1.43–4.04%), *Deltaproteobacteria* (1.44–2.98%), *Cytophagia* (1.25–2.93%), *norank_Candidatus_Saccharibacteria* (1.01–2.58%), and *Acidobacteria_Gp3* (1.09–1.99%) also have high relative abundances in four replicates. The soil bacteria community relative abundances in SMO20 group (with 152.44 mg/kg Hg^2+^) are shown in Figure 6B. *Alphaproteobacteria* (15.82–18.43%), *Sphingobacteriia* (6.95–11.26%), *Gammaproteobacteria* (6.71–9.25%), *Deltaproteobacteria* (3.45–9.19%), *Betaproteobacteria* (5.37–6.83%), *Acidobacteria_Gp6* (4.61–5.73%), *Anaerolineae* (2.41–6.33%), and *Planctomycetia* (2.96–4.76%) have very high relative abundances in four replicates. *Flavobacteriia* (1.24–7.58%), *Acidobacteria_Gp4* (2.62–2.98%), *Gemmatimonadetes* (1.17–3.18%), *Actinobacteria* (1.39–2.97%), and *Acidobacteria_Gp3* (1.53–2.13%) have high relative abundances in four replicates. The soil bacteria community relative abundances in SMO30 group (with 63.48 mg/kg Hg^2+^) are shown in Figure 6C. *Alphaproteobacteria* (15.82–18.43%), *Acidobacteria_Gp4* (7.74–10.89%), *Sphingobacteriia* (4.51–10.92%), *Acidobacteria_Gp6* (6.20–8.45%), *Actinobacteria* (4.14–10.09%), *Betaproteobacteria* (4.17–7.25%), *Gammaproteobacteria* (5.17–6.05%), *Deltaproteobacteria* (4.14–5.95%), *Planctomycetia* (2.80–6.42%), and *Spartobacteria* (2.35–5.72%) have very high relative abundances in four replicates. *Subdivision3* (1.84–3.30%), *Acidobacteria_Gp3* (1.96–2.71%), and *norank_candidate_division_WPS-1* (1.10–3.99%) have high relative abundances in four replicates. The soil bacteria community relative abundances in SMO500 group (with 59.77 mg/kg Hg^2+^) are shown in Figure 6D. *Alphaproteobacteria* (16.39–29.83%), *Sphingobacteriia* (7.74–12.88%), *Acidobacteria_Gp4* (4.12–10.44%), *Betaproteobacteria* (5.04–8.63%), *Gammaproteobacteria* (1.70–7.82%), *Acidobacteria_Gp6* (3.53–6.34%), *Actinobacteria* (3.12–8.04%), *Anaerolineae* (1.66–6.83%), *Gemmatimonadetes* (2.71–6.80%), *Planctomycetia* (2.29–3.23%), and *Acidobacteria_Gp3* (2.43–3.17%) have very high relative abundances in four replicates. The soil bacteria community relative abundances in SMO650 group (with 109.44 mg/kg Hg^2+^) are shown in Figure 6E. *Betaproteobacteria* (7.95–42.90%), *Alphaproteobacteria* (10.72–27.51%), *Bacteroidia* (0.25–36.35%), *Sphingobacteriia* (0.90–9.33%), *Acidobacteria_Gp1* (0.51–8.88%), *Actinobacteria* (1.44–5.49%), and *Gammaproteobacteria* (1.59–6.52%) have very high relative abundances in four replicates. The within-group analysis results revealed significant differences in the structures of the soil bacterial communities in four replicates of the same group. Further, the same soil bacterial community has significantly different relative abundances of taxa in four replicates of the same sample group, especially in SMO650 group.

The soil bacterial community relative abundances in five groups (different Hg^2+^ contents) are shown in Figure 6F. *Alphaproteobacteria* (17.21–23.38%), *Betaproteobacteria* (6.15–22.30%), *Sphingobacteriia* (4.56–9.86%), and *Gammaproteobacteria* (4.03–12.40%) have extremely high relative abundances in five groups. *Acidobacteria_Gp4* (1.46–8.79%), *Acidobacteria_Gp6* (1.10–7.11%), *Actinobacteria* (2.03–6.40%), *Deltaproteobacteria* (1.38–7.28%), *Planctomycetia* (0.76–4.06%), and *Acidobacteria_Gp3* (1.59–2.89%) also have high relative abundances in five groups. These inter-group analysis results indicated that both the same and different taxa dominated the bacterial communities in five different groups. The same dominant bacterial community has significantly different relative abundances of taxa in five different groups (different Hg^2+^ contents) at the class level.

### 3.6 The relationships between the soil environmental factors and the soil bacterial community structure for the five groups at the phylum and class levels

**FIGURE 7:**
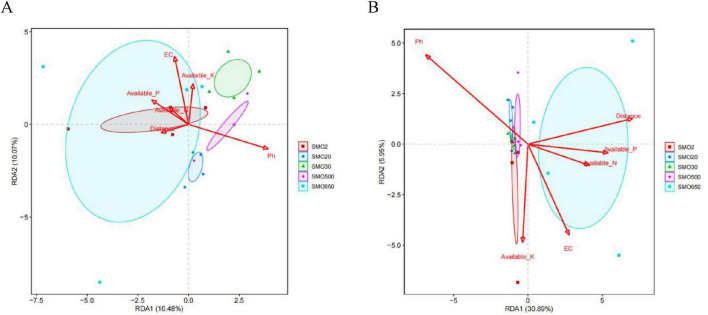
**(A)** The redundancy analysis (RDA) between soil environmental factors and soil bacterial community structure for five groups at the phylum level. **(B)** The RDA between soil environmental factors and soil bacterial community structure for five groups at the class level [R vegan package (version 2.5-6) analysis].

The results of the redundancy analysis (RDA) indicate that the soil environmental factors together explained 26.55% of the total variation in the soil bacterial community structure at the phylum level, and explained 36.84% of it at the class level (Figures 7A, B). At the phylum level, the pH was negatively correlated with the other five soil environmental factors. In terms of the effect of six soil environmental factors on the bacterial community structure, the pH and EC played the paramount role in shaping the soil bacterial community structure of five different groups. At the class level, the pH was also negatively correlated with the other five soil environmental factors. The pH and distance factor (mercury content) played the crucial role in the soil bacterial community structure of the five different groups.

## 4 Discussion

Once soil is contaminated by mercury, the mercury in the soil is very easily transferred to the roots, stems, and leaves of plants through plant enrichment, which seriously affects the normal growth and development of the plants (He and Peng, 2023; Wang et al., 2023). In addition, the mercury in soil seriously affects the stability of soil ecosystems, especially the composition and spatial distribution of soil microorganisms (Liu et al., 2018; Egbo et al., 2021). Previous researches has confirmed that the Zn, Cd, Cu, and Pb concentrations of soil are closely correlated with the microbial biomass (Wang et al., 2003) and that different microbial communities have different tolerances to stress imposed by different heavy metals (Zhao J. et al., 2014). The negative effect of copper on bacterial community diversity of mine drainage from the copper mines has been attributed to the limitations of microbial metabolism and other functions, culminating in reduced soil microbial diversity. However, the effects of heavy metal types and amounts on microbial diversity are different (Li T. et al., 2024).

In this study, analysis of the bacterial community diversity of soil samples with different mercury contents near a mercury mining area revealed that, at both phylum and class levels, the total numbers of OTUs in the high mercury content soil sample groups (SMO2, SMO20, and SMO650) are greater than those in the low mercury content soil sample groups (SMO30 and SMO500). Our results are inconsistent with those of Frey and Rieder (2013), Frossard et al. (2017), and Shan et al. (2016). We speculate that the reasons for the bacterial community diversity differences are mainly related to the internal environmental factors of the soil, such as the pH, EC, distance (mercury content), AN, AP, AK, and other factors. Most previous studies mainly simulated the mercury contamination environment in the laboratory, such as those conducted by Wang (2017), Jennifer et al. (2009), and Xie et al. (2011). In contrast, we collected samples of natural soils near a mercury mining area to account for the fact that heterogeneity of the soil environment can easily result in different effect on the soil bacterial community diversity. Our test environment and results were relatively close to those of Liu et al. (2014). The survival of a large number of different bacterial communities provides evidence of long-term adaptation to the high mercury stress environment. Maliszewska et al. (1985) confirmed that Cu and Hg compounds significantly affect microbial proliferation.

Through alpha diversity analysis of the bacterial communities in our samples using the number of Reads, the OTUs number, the Shannon and Simpson indexes, we determined that there were large differences in the diversities of the bacterial communities in five groups, except for the Simpson index. The diversities of the bacterial communities in SMO2 and SMO20 (high mercury contents) were significantly higher than those in SMO30, SMO500 (low mercury contents), and SMO650 groups (moderate mercury content), which suggests that in our chosen outdoor natural environment, the bacterial community diversity was affected by many internal factors of the soil. These many internal factors of the soil eventually led to differences in the numbers of unique bacterial communities in the five groups. In addition, this result indicates that there is large heterogeneity among the five soil sample groups collected near the mercury mining area.

Soil contains a plethora of microorganisms, namely bacteria, fungi, and actinomycetes, which collectively play an irreplaceable role in maintaining the stability of soil ecosystems. The quantity and composition of microorganisms in soil are mainly affected by environmental factors, such as soil nutrients, soil aeration, pH, and heavy metal contents (Yang et al., 2023). When soil is contaminated with heavy metals, the composition and structure of soil microorganisms will change to some extent, and since soil microorganisms can objectively reflect shifts in key properties of soil quality, they are often used as important biological indicators of altered soil environment quality. Moreover, compared with fungi and actinomycetes, bacteria are more sensitive to heavy metal pollution (Li et al., 2004). Mercury in soil inhibits the growth of bacteria, fungi, and actinomycetes communities, which also affects activity of bacteria and their community structure. However, there are differences in how microorganisms respond to soil mercury stress. The bacterial abundance was significantly correlated with the soil organic matter content rather than the total Hg (THg) concentration (Liu et al., 2014). One study found no effects of 0–1 mg/kg of mercury in forest soil whereas 20 mg/kg of mercury significantly altered the diversity and genetic structure of its soil microbial community (Jennifer et al., 2009). The relative abundance of Nitrospirae decreased, whereas that of Gemmatimonadetes increased significantly along the increasing soil THg and MeHg concentrations (Liu et al., 2014). In soils treated with at least 3.2 μg Hg g^–1^ dry soil, the basal respiration of bacteria was strongly affected. High bioavailable Hg also caused significant changes in the bacterial T-RFLP profiles. Members of the *Alphaproteobacteria* (*Rhodospirillales*) and *Betaproteobacteria* (*Burkholderiales*) were found to be Hg-tolerant (Frey and Rieder, 2013). The highest concentration of Hg (32 μg Hg g^–1^ dry soil) caused severe diversity loss and shifts in the bacterial community structures and composition. Lower concentrations of Hg (≤ 3.2 μg Hg g^–1^ dry soil) had only a limited effect on the soil microbiome. Fungal communities were generally less affected than bacterial communities (Frossard et al., 2017).

In this study, by comparing and analyzing the distributions and compositions of predominant taxa in the bacterial community within each group and among the groups of soil samples, we found the same and different predominant taxa in their composition at the phylum and class levels. *Proteobacteria* (39.82–45.40%), *Acidobacteria* (14.45–16.17%), and *Bacteroidetes* (13.71–15.07%) were the most representative dominant bacterial communities in the five groups at the phylum level. *Alphaproteobacteria* (17.21–23.38%), *Betaproteobacteria* (6.15–22.30%), *Sphingobacteriia* (4.56–9.86%), and *Gammaproteobacteria* (4.03–12.40%) were the most representative dominant bacterial taxa in five groups at the class level. This finding suggests that heterogeneity of internal factors of the soil sample groups resulted in different effects on the structures of their bacterial communities. The different predominant bacterial taxa in five groups of soil samples exhibited differential tolerances to mercury exposure (content as a heavy metal stress) and other soil environmental factors. The main reasons for this seems to be related to the heterogeneity of the internal soil factors (Zhu et al., 2021; Osterwalder et al., 2019; Dranguet et al., 2019). This result is consistent with that of Prasad (2013) and Liu et al. (2014).

The survival of abundant amounts of these dominant members of the soil bacterial community indicates that they have strong adaptabilities to tolerate the stress caused by the high mercury content soil environment near the mercury mining area. This is compelling evidence of their high degree of adaptation to the existing soil environment around the studied mercury mining area. Furthermore, the survival of the different dominant bacteria within each group and between the sample groups confirms that inconsistent responses of differing bacterial taxa are highly related to the heterogeneity of the many internal factors of the soil environment (Zhu et al., 2021; Signorini et al., 2021), such as its mercury content (heavy metal stress). Our results are quite similar to those of Feris et al. (2003) (the Clark Fork River in western Montana, United States), Gillan et al. (2005) (the Sørfjord in southern Norway), and Liu et al. (2014) (in paddy soils in China). Our results and the results of these previous studies provide insights into the distribution patterns of bacteria communities along long-term mercury-contamination gradients in soils around mercury mining areas.

The structures of the soil microbial communities in the soil samples were obviously affected by the internal environmental factors of the soil, such as the cycle of soil nutrients, pH, EC, and changes in soil heavy metals (Mbuthia et al., 2015; Mija et al., 2021). Mercury contamination, which is regarded as a type of environmental stress, usually plays a crucial role in decreasing the microbial diversity and altering the community structure of soil bacteria. To further explore the relationships between the bacterial community structure and soil environmental factors, we utilized the RDA method to conduct a more in-depth analysis. Huang et al. (2024) confirmed that the soil pH strongly influenced bacterial and fungal beta diversity compared with climate, soil nutrients, and plant properties. Our results indicate that the pH, EC, distance (Hg^2+^ content), AP, AK, and AN (Zhu et al., 2021) affected the distribution of the bacterial communities at the phylum and class levels. In particular, at the class level, the pH, distance (Hg^2+^ content), and EC had important effects on the structures of the bacterial communities. This result is consistent with that of Shi et al. (2021), where soil pH plays a pivotal role in shaping microbial diversity and community composition in terrestrial ecosystems. Meanwhile, these results also suggest that the dominant taxa in the sampled bacterial communities are better able to adapt to long-term mercury-contamination of their soil environment (Dranguet et al., 2019). In addition, our results indicate that the mercury content of the SMO2, SMO20, SMO30, and SMO500 gradually decreases with the increase of the distance from the sewage outlet, the SMO650 group is the farthest from the sewage outlet, but mercury content reaches a moderate level. We preliminarily speculate that this may be related to the distribution of discontinuous mercury mines under shallow layers soil. Under moderate mercury content level, the number of OTUs of SMO650 close to that of SMO2 and SMO20 at both phylum and class levels, this result indicate that SMO650 group has relative high bacterial community diversity, it is also an evidence of the adaptability of different mercury resistant bacteria to soil environment. Try to isolate functional microorganisms with mercury resistance from these highly adaptive microbial communities, and select and breed strains with nitrogen fixation, phosphorus solubilization or heavy metal passivation capabilities for use in mercury-contaminated soil remediation agents. Or further encapsulate these excellent strains into capsules to make sustained-release agents, which will have considerable application potential in the vegetation restoration of mercury-contaminated areas or the cultivation of non-food crops. This is also one of our main research contents in the future.

## 5 Conclusion

From the perspective of mercury contamination’s impact on soil bacterial communities under natural long-term mercury-contamination soil environment, our experimental results lead us to infer the following. (1) At the same taxonomic level, the soil bacterial community diversity in the SMO2, SMO20, and SMO650 sample groups (relatively high Hg^2+^ contents) are higher than those in the SMO30 and SMO500 sample groups (relatively low Hg^2+^ contents); (2) At both phylum and class levels, the same dominant taxa in the bacterial community have different relative abundances among the five sample groups. (3) The diversity and structure of the soil bacterial community is highly correlated with soil environmental factors, being critically influenced by the pH, distance (Hg^2+^ content), and EC.

## Data Availability

The datasets presented in this study can be found in online repositories. The names of the repository/repositories and accession number(s) can be found below: https://ngdc.cncb.ac.cn/gsa/browse/CRA011955, GSA: CRA011955.
